# Enhanced photosynthetic output via dichroic beam-sharing

**DOI:** 10.1007/s10529-012-1021-5

**Published:** 2012-08-30

**Authors:** Mark D. Redwood, Raveen Dhillon, Rafael L. Orozco, Xu Zhang, David J. Binks, Mark Dickinson, Lynne E. Macaskie

**Affiliations:** 1 Unit of Functional Bionanomaterials, School of Biosciences, University of Birmingham, Edgbaston, Birmingham, B15 2TT UK; 2 School of Physics and Astronomy & Photon Science Institute, University of Manchester, Manchester, M13 9PL UK; 3 State Key Laboratory of Bioreactor Engineering, East China University of Science and Technology, 130 Meilong Road, Shanghai, 200237 People’s Republic of China

**Keywords:** *Arthrospira* (*Spirulina*) *platensis*, Bioenergy, Biohydrogen, Biofuel, Dichroic beam-sharing, *Rhodobacter sphaeroides*

## Abstract

Microbial solar biofuels offer great promise for future sustainable food, fuels and chemicals but are limited by low productivities and a requirement for large land areas to harvest sunlight. A 71 % increase in combined photosynthetic activity was achieved by illuminating both *Rhodobacter sphaeroides* and *Arthrospira* (*Spirulina*) *platensis* from a single beam of simulated sunlight, divided using a dichroic mirror. Therefore, this technique is termed ‘dichroic beam-sharing’, in which the complementary action spectra of two different useful micro-organisms, belonging to green and purple groups, is exploited and allows a single beam of sunlight to be shared efficiently between separate photobioreactors. Because the action spectra of these two organisms are typical of large groups, this novel method could increase the productivity of photosynthetic micro-organisms in the production of diverse commodities.

**Electronic supplementary material:**

The online version of this article (doi:10.1007/s10529-012-1021-5) contains supplementary material, which is available to authorized users.

## Introduction

**Fig. 1:**
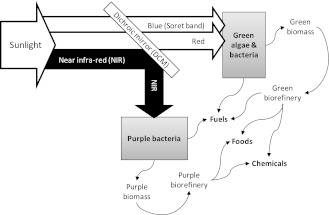
Dichroic beam-sharing (conceptual illustration). A dichroic mirror divides the solar spectrum between different organisms having complementary action spectra, such as green algae and purple bacteria. Note that algal photosynthesis, while capturing CO_2_, is oxygenic whereas anoxygenic photosynthesis by purple bacteria is anaerobic and H_2_ production is strongly inhibited by O_2_, hence co-culture is not practical (see text)

Despite the concerns over fossil fuels (Kerr 2011), the adoption of biofuels is still limited, primarily by photosynthetic efficiency (PE). For crop plants, PE is just 0.5–1 % (Archer and Barber 2004) which, if biofuels are to replace fossil fuels so atmospheric carbon is stabilised by 2100, would necessitate a doubling of agricultural land area (Gurgel et al. 2008). In contrast, photosynthetic microorganisms can produce biofuels with a significantly greater PE (up to 9 % e.g. Guo et al. 2011) and can be cultivated in areas where crops cannot, such as on steep slopes, contaminated land, buildings or water, and provide a range of valuable co-products (Fig. 1) (Blankenship et al. 1995; Metzger and Largeau 2005). However, these benefits are currently offset by the cost of photobioreactors and so further improvements in PE are required for microbial solar biofuels to become viable. Attempts to enhance PE biochemically have, so far, met with limited success (e.g. Work et al. 2012 and references therein). In this study we demonstrate a complementary approach: dichroic beam-sharing.

Oxygenic photosynthetic microorganisms (including cyanobacteria, algae and chloroplasts; usually green) and anoxygenic purple non-sulfur bacteria have complementary action spectra and so their co-cultivation has the potential to improve overall photobioreactor efficiency. A co-culture of these is not suitable because oxygen generated by the green microorganisms inhibits the purple, and a bi-layer approach (Miyamoto et al. 1987) is limited by light scattering and absorption in the upper layer reducing light penetration to the lower. However, a dichroic mirror (DCM) has a reflection spectrum, *R*(λ), (simply related to the transmittance spectrum, *T*(λ) = 1−*R*(λ)) that can be engineered by control of its surface coating and so can be used to direct to each micro-organism its favoured part of the solar spectrum (Fig. 1). In this study, we demonstrate this approach for *Arthrospira* (*Spirulina*) *platensis* (a foodstuff; Raoof et al. 2006) and *Rhodobacter sphaeroides* ZX-5 (a hydrogen producer; Redwood et al. 2012a) and show that up to 100 % increase in combined PE could be achieved.

## Methods

### Solar simulator

**Fig. 2:**
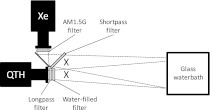
Close-match solar simulator. Parts and configuration. Xe: Xenon lamp (LOT Oriel P/N LSO104; 150 W). Shortpass filter: Edmund Optics (P/N NT64-668), variable cut-off: 750 nm at 45°; QTH: quartz tungsten halogen lamp (Oriel P/N 60000; 100 W); longpass filter: Thorlabs P/N FGL780; water filter: glass petri dish filled with deionised water and sealed with a rubber gasket; 7 mm depth. The positions, when in use, of two identical dichroic mirrors (Thorlabs P/N DMLP638L) are marked by X

Xenon lamps are commonly used as solar simulators because their emission spectrum resembles that of the sun over most of the spectrum. This match is often further improved using specially designed ‘AM1.5’ filters. The American Society for Testing and Materials (ASTM) define a solar simulator as ‘class A’ if its emission spectrum differs from the solar spectrum by no more than 25 % over all of several non-equal bands. Filtered xenon lamps are class A over the visible and near-infrared region (400–1,000 nm) as a whole but are only class B (<40 % difference) between 800 and 900 nm because in this region they emit several strong spectral lines absent from the solar spectrum. The poorer spectral match in this region is not significant for many applications but is relevant to the current study because this region corresponds to the most important part of the action spectrum for purple non-sulfur bacteria. Hence, to ensure a good spectral match in this region, a filtered xenon lamp (LOT Oriel P/N LSO104; 150 W) was combined with a quartz tungsten halide (QTH) lamp (Oriel P/N 60000; 100 W), the emission spectrum of which is much closer to that of sunlight in the 800–900 nm spectral region. The spectrum of the Xe lamp was filtered to remove wavelengths longer than 725 nm whilst that of the QTH lamp was filtered to remove wavelengths shorter than 725 nm. A water-filter further improved the spectral match in the near-infrared by mimicking the absorbance of atmospheric H_2_O. The beams from each lamp were overlaid (Fig. 2) to produce a combined field of illumination that was a close match to the solar spectrum, including in the 800–900 nm region important for this study. The spectra of the xenon lamp, the combined lamps and the solar spectrum were measured and compared using a spectrometer (Ocean Optics USB4000 VIS–NIR with Spectrasuite software in relative irradiance mode) calibrated using a blackbody source traceable to the UK National Physical Laboratory. The intensity of the combined light field could be adjusted by focusing or defocusing the beams from the lamps and was set to produce an intensity of 10 W/m^2^, as measured by a PAR sensor (thermopile; Skye, UK, 400–1,000 nm), over an area of 750 cm^2^. This intensity was chosen so that any light saturation effects could be neglected. Light limitation was confirmed practically; activity was proportionate to light intensity in the range 2.5–25 W/m^2^ (see Supplementary Fig. 1). Two identical dichroic mirrors (Thorlabs P/N DMLP638L) were used to spectrally divide the combined-lamp illumination so that the transmitted light was incident onto *A.* *platensis* whilst the reflected light was incident onto *R.* *sphaeroides*.

### Strains and culture conditions

*Arthrospira* (*Spirulina*) *platensis* (strain no. 86.79) was purchased from Sammlung von Algenkulturen der Universität Göttingen and maintained in 400 ml Raoof’s low-cost medium (Raoof et al. 2006) at 30 °C with continuous mechanical mixing and illumination (9 W fluorescent lamp; 14 W/m^2^) in a 1 l water-jacketed glass vessel. 300 ml of the culture (75 % of total) was discarded weekly and replaced with fresh Raoof’s medium. Chlorophyll was measured spectrometrically according to Raoof et al. (2006) and found to be proportional to dry weight (DW) and the OD_660_ during the first 7 days of cultivation, after which, the chlorophyll concentration declined while DW continued to increase.

*Rhodobacter sphaeroides* ZX-5 was cultured as described previously (Redwood et al. 2012b), using sodium butyrate (30 mM) as the primary carbon source. To provide consistent stocks for H_2_ production assays a single batch of *R.* *sphaeroides* cells was divided and aliquots were preserved at −80 °C. Cells were grown for 72 h (30 °C, intermittent manual shaking) in completely filled bottles under 75 W/m^2^ from a 300 W halogen lamp, harvested by centrifugation (4,000×*g*, 15 min) and resuspended in butyrate medium with 15 % (v/v) glycerol, before freezing in liquid N_2_.

Experiments used a glass waterbath (30 °C) receiving illumination at 10 W/m^2^ from below by the solar simulator, in which reactors (12 ml total internal volume) were positioned in the beam. For *A.* *platensis* growth tests, inocula were taken from an actively growing maintenance culture, diluted to an OD_660_ of 0.04 (25 mg DW/l) with fresh medium, transferred (8 ml) into reaction vials and incubated under irradiance from the solar simulator with continuous mixing by bubbling with moistened air through 18G needles. OD_660_ was recorded after 48 h. For *R.* *sphaeroides* H_2_ production tests, aliquots were thawed and diluted to 1,000 mg DW/l (OD_660_ = 0.302) with fresh butyrate medium, then dispensed (4 ml) into reaction bottles (12 ml internal volume), sealed with anaerobic stoppers, purged with argon (30 min) and incubated under irradiance from the solar simulator. The H_2_ concentration in the headspace was measured after 36 h as described previously (Orozco et al. 2012).

## Results

**Fig. 3:**
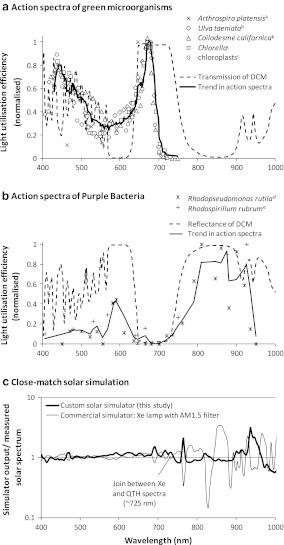
Photosynthetic action spectra of green microorganisms (**a**) and purple bacteria (**b**) in relation to transmission and reflectance properties of a DCM, and a comparison of the spectral match of a commercial solar simulator with the one constructed for this study (**c**). Action spectra were compiled from ^a^Chen et al. (2010), ^b^Haxo and Blinks (1950), ^c^Chen (1952), ^d^Nogi et al. (1985), ^e^French (1937) and converted using Engauge Digitizer v4.1. Values from all organism-sets were pooled and trendlines were drawn by smoothing by 5 % (128 points in (**a**) smoothed by 6; 36 points in (**b**) smoothed by 2). The simulator spectra shown in (**c**) are relative to a solar spectrum measured at noon on a clear June day in Manchester (UK) with the same spectrometer and configuration; proximity to 1 indicates spectral match. AM1.5G: Manufacturer’s filter optimised for spectral match at ground level, 48° N. The AM1.5 filtered xenon lamp conforms to the highest current standard for spectral match (ASTM 2010) but would be unsuitable for optical studies of purple bacteria due to the poor match in the 800–900 nm band (see text)

Figure 3a, b compare the action spectra of green micro-organisms and purple bacteria with the transmission and reflectance spectra of the dichroic mirror (manufacturer’s data) respectively, showing a good match in both cases. Manufacturer’s data were confirmed (Supplementary Fig. 2). The spectra of the filtered xenon lamp and the combined lamps relative to the solar spectrum are given in Fig. 3c, and show an improved spectral match in the 800–900 nm region, now class A rather than B according to ASTM classification.

**Fig. 4:**
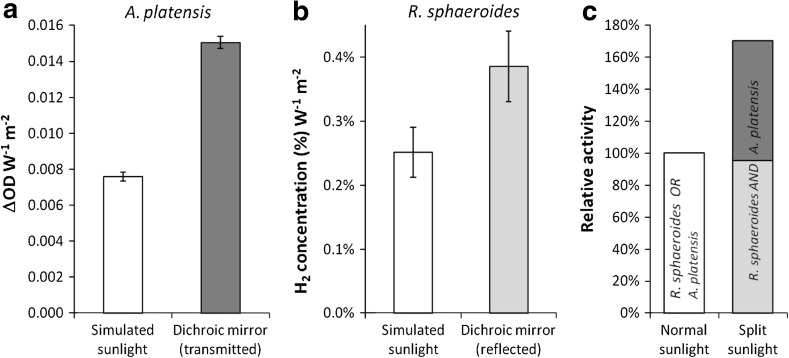
Photosynthetic activity of *A.* *platensis* (**a**) and *R.* *sphaeroides* (**b**) and combined activity from a single beam of ‘sunlight’ (**c**). In (**a**) and (**b**), activities are expressed per unit of light intensity; the reflected and transmitted intensities were, 6.2 and 3.8 W/m^2^, respectively. In (**c**), the *stacked dark* and *pale bars* represent the combined activities of *A.* *platensis* under transmitted sunlight (*dark*) and *R.* *sphaeroides* under reflected sunlight (*pale*), relative to either singly under complete sunlight (shown as a *white bar* for comparison). **a** Shows means and standard errors from four independent experiments with and without the dichroic mirror each including 6 replicates. **b** Shows four independent experiments with the mirror and three in ‘sunlight’, each including 4 replicates. ΔOD: increase in OD_660_

Application of the dichroic mirror reduced the light supplied to *A.* *platensis* (transmitted light) by 62 % but the growth only by 25 %. The difference was statistically significant (*t* test, *P* < 5 %). Figure 4a shows that, in relation to the total light intensity, the cultures with transmitted light were twice as efficient as those using a direct beam. Conversely, for H_2_ production by *R.* *sphaeroides* the dichroic mirror reduced the supplied light (reflected) by 38 % causing no significant difference in photosynthetic activity with an average productivity equivalent to 95 % of the control (full spectrum). Figure 4b shows that, in relation to the total light intensity, the cultures with reflected light were 1.5 times as efficient as those using a direct beam. Figure 4c shows that the combined photosynthetic activity was equivalent to 170 % in comparison to either reactor individually.

## Discussion

The dichroic mirror provided almost complete reflection of blue and NIR wavelengths corresponding to the action maxima of purple bacteria (Fig. 3) and, accordingly, the mirror caused no detectable reduction in photosynthetic activity. Similarly, the mirror provided almost complete transmission of red wavelengths corresponding to the action maximum of green organisms. However, ~50 % of light was lost through reflection in the 400–550 nm band, which represents ~50 % of the total action for green organisms. Therefore, the overall reduction in active radiation was ~25 % which corresponds to the observed 25 % reduction in growth for *A.* *platensis*. Hence, the observed 25 % reduction in activity is attributed to the partial reflection of useful ‘blue’ (400–550 nm) light. Further mirror development of dichroic mirrors would aim to increase transmission in the ‘blue’ band, potentially increasing the combined photosynthetic activity from 170 % towards the limit of 200 %.

The present study demonstrates the biological compatibility of dichroic beam-sharing with one pair of useful organisms. However, the action spectrum of *A.* *platensis* is typical of green micro-organisms of diverse genera and the chloroplasts of higher plants (Fig. 3), and so this technique should be applicable to other organisms with useful products. Further work is also required to establish to what degree the additional cost of using this technique would offset the benefit of doubled overall PE, set against food security and land use issues which may ultimately prove a critical socio-economic factor. This additional cost is likely to be minimised for photobioreactor designs utilising optical fibre light-delivery (Erickson et al. 2011) which would require only small dichroic mirrors. The optical damage threshold of the mirrors is typically very high (>1,000 MW/m^2^; manufacturer’s specification for the mirror used in this study), three orders of magnitude greater than the peak solar intensity at a concentration factor of 1,000.

## Conclusion

Dichroic beam-sharing offers a practical opportunity for increasing photosynthetic activity by up to 100 %. This study demonstrated a 71 % increase in the combined photosynthetic activity of *A.* *platensis* and *R.* *sphaeroides*, which have been studied in the production of sustainable food and H_2_ fuel, respectively. Losses were associated mainly with *A.* *platensis* and attributed to the partial reflection of useful ‘blue’ (400–550 nm) light by the dichroic mirror. The demonstrated principle indicates that solar photosynthetic activity could be increased without requiring additional land or sunlight, using existing technology to enhance the sustainable production of foods, fuels and chemicals via duplexed photobioreactor systems.

## Electronic supplementary material

Below is the link to the electronic supplementary material.

Supplementary material 1 (DOCX 247 kb)
