# Supplementary material for: Enhanced photosynthetic output via dichroic beam-sharing
Source: Biotechnol Lett. 2012 Aug 30;34(12):2229–34. doi: 10.1007/s10529-012-1021-5 (PMC3487006; doi:10.1007/s10529-012-1021-5)
Supplement: Supplementary file 1 — Supplementary material 1 (DOCX 247 kb) [file 10529_2012_1021_MOESM1_ESM.docx]

Supplementary Figure 1


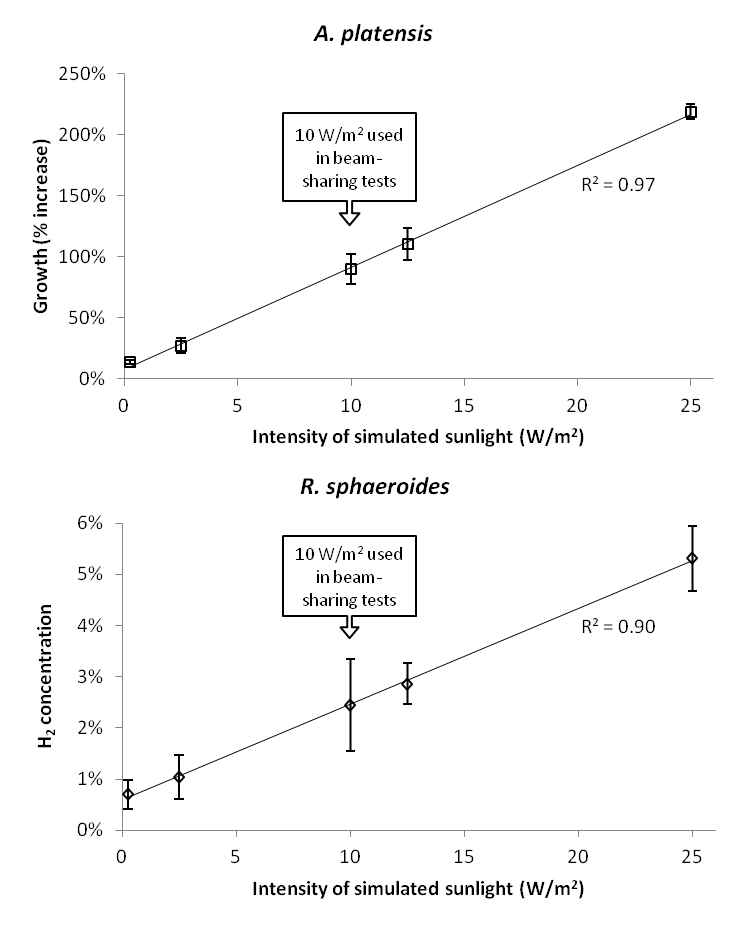


Legend to Supplementary Information 1

Light limitation experiments were carried out as described in Methods. Alternative light intensities were obtained by condensing the beams of the solar simulator and by filtering sections of the field using neutral density filters of varying transmittance. Error bars indicate standard deviations. Reported light saturation points for *A. platensis* and *R. sphaeroides* are in the range 40‑400 W/m^2^ (e.g. Converti et al, 2006, Biochem Eng J 32:13 and Gadamshetty et al, 2008, IJHE 33:2138, respectively).

Supplementary Information 2


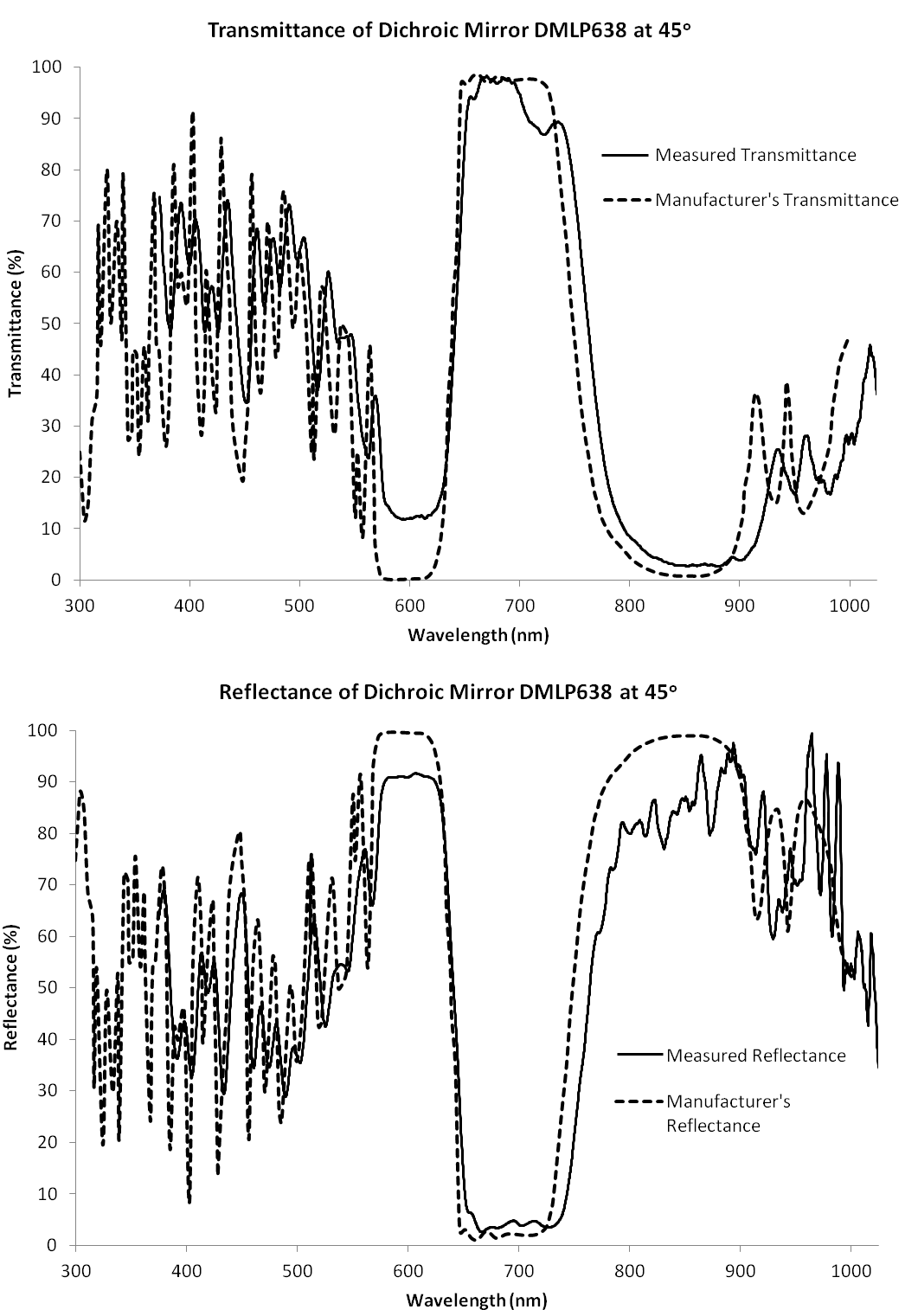


Legend to Supplementary Information 2

R and T data measurements were made using an Ocean Optics USB4000 VIS-NIR spectrometer. The spectra shown in Fig. 3 were drawn from manufacturer’s data. Dichroic Mirror DMLP638 was purchased from Thorlabs.
